# Comparative Anticonvulsant Efficacy of Natural, Artificial and Cultivated Bovis Calculus in PTZ-Induced Acute Seizures

**DOI:** 10.3390/biomedicines14081727

**Published:** 2026-07-31

**Authors:** Donghua Yu, Jing Wang, Mingxia Xie, Youyuan Lu, Hanqing Wang

**Affiliations:** 1 College of Pharmacy, Ningxia Medical University, Yinchuan 750004, China; m15156575298@163.com (D.Y.); m15735213692@163.com (J.W.); 2 College of Traditional Chinese Medicine, Hunan University of Chinese Medicine, Changsha 410007, China; xiemingxia0618@163.com; 3 Ningxia Regional Characteristic Traditional Chinese Medicine Collaborative Innovation Center Co-Constructed by the Province and Ministry, Ningxia Engineering and Technology Research Center for Modernization of Regional Characteristic Traditional Chinese Medicine, Ningxia Medical University, Yinchuan 750004, China

**Keywords:** Bovis Calculus, Bovis Calculus Artifactus, anti-seizure, neuroprotective, neuroinflammatory, neurotransmitters

## Abstract

**Background:** Bovis Calculus (NBC) is widely used for the management of convulsive disorder, but its clinical application is constrained by limited availability and high cost. Bovis Calculus Artifactus (BCA) and Bovis Calculus Sativus (BCS) are commonly employed as substitutes. However, systematic evidence comparing their anticonvulsant efficacy and pharmacological characteristics remains limited. Methods: This study evaluated the substitution potential of BCA and BCS relative to NBC and characterized their distinct pharmacological profiles in a pentylenetetrazol (PTZ)-induced acute seizure mouse model. Seizure latency and duration were evaluated, hippocampal pathology was examined by hematoxylin–eosin and Nissl staining, neurotransmitters and neuroinflammatory factors were quantified. Network pharmacology and molecular docking analyses were used to explore the underlying mechanisms. **Results:** The results showed that preventive administration of NBC, BCA, and BCS significantly alleviated PTZ-induced acute seizures, although their efficacy patterns differed markedly. NBC exhibited the broadest anticonvulsant spectrum, significantly prolonging seizure latency at multiple seizure stages. BCS showed a more restricted effect, primarily delaying stage IV seizures, whereas BCA predominantly reduced seizure frequency without significantly affecting seizure latency, suggesting that seizure frequency may represent a sensitive endpoint for evaluating certain anticonvulsant agents. Histopathological analyses demonstrated that all three preparations exerted neuroprotective effects in hippocampal subregions. NBC provided the most extensive morphological preservation in H&E staining, while all treatments significantly improved neuronal survival as assessed by Nissl staining. Biochemical analyses revealed distinct regulatory characteristics among the three Bovis Calculus sources. All treatments partially restored PTZ-induced neurotransmitter disturbances and attenuated neuroinflammatory responses. BCA showed prominent regulation of neurotransmitters and IL-1β, NBC exhibited broader modulation of neurotransmitter homeostasis, whereas BCS displayed stronger suppression of IL-6 and TNF-α despite limited effects on 5-HT regulation. These findings indicate that comparable protective effects against PTZ-induced acute seizures may be achieved through different neurochemical and inflammatory regulatory patterns. Network pharmacology and molecular docking analyses further suggested the potential involvement of PI3K-AKT and MAPK-related signaling pathways in the effects of NBC and its substitutes. **Conclusions:** Collectively, this study provides a comparative evaluation of NBC, BCA, and BCS and indicates that their protective activities against PTZ-induced seizures are associated with distinct neurochemical and anti-inflammatory profiles. These findings offer experimental evidence supporting the rational evaluation and application of alternative Bovis Calculus resources.

## 1. Introduction

Epilepsy is one of the most common and disabling chronic neurological diseases, afflicting nearly 65 million people worldwide [1]. It was initially recognized by seizures and convulsions, and is characterized by recurrent spontaneous seizures [2]. Seizure activity can trigger or accelerate adverse neurological sequelae, causing disability and mortality [3]. To prevent seizure recurrence, patients typically take anti-seizure drugs long-term [4]. Although numerous novel anti-seizure drugs have been developed, over 30% of patients still suffer from seizures due to pharmacoresistance and inadequate treatment [5,6,7]. Moreover, most commonly used anti-seizure drugs are associated with extensive neurotoxic adverse effects, leading to medication nonadherence or exacerbation of behavioral comorbidities [1]. Accumulating evidence demonstrates that neuroinflammation, neuronal damage, and oxidative stress are associated with seizure generation [2,8,9]. The complexity of seizure disorders underscores the urgent need for multi-targeted therapeutic strategies.

Traditional Chinese medicine (TCM) has been used to treat convulsion and epilepsy for thousands of years [10]. The fundamental principles of TCM theory are centered on achieving a harmonious equilibrium between Yin and Yang, as well as the five elements within the body. By utilizing herb medicines and other therapeutic methods, TCM aims to restore balance and eliminate disease [11]. For instance, Gastrodiae Rhizoma (Tianma) and Uncariae Ramulus Cum Uncis (Gouteng) are often combined to treat convulsive disorders [12]. Ganoderma (Lingzhi) powder can reduce seizure frequency and severity per episode [13]. Scorpio (Quanxie) demonstrates significant anticonvulsant effects, characterized by sustained pharmacological activity [14].

Bovis Calculus (Niuhuang, NBC) has been used as a traditional Chinese medicine for more than 2000 years [15]. It has been traditionally used to treat a range of disorders, including clearing heart fire, resolving phlegm, promoting resuscitation, cooling the liver, extinguishing wind, and detoxifying [16]. Pharmacological studies have demonstrated that NBC exhibits potent activities, including anti-inflammatory, anti-cerebral ischaemic injury, anticonvulsant, and other effects [17]. Due to limited natural sources and high price, Bovis Calculus Artifactus (BCA) and Bovis Calculus Sativus (BCS) have been included in the Chinese Pharmacopoeia as substitutes for Bovis Calculus to meet the clinical needs [18]. In addition, Bovis Calculus Culture (BCC) was approved in 1972 as a substitute for NBC to address clinical demand. They are composed of bovine bile, cholic acid, taurine, bilirubin, cholesterol, and trace elements [18]. Although the main chemical compositions are similar, their metabolic characteristics differ [19], which may lead to different pharmacological effects. For instance, it has been reported that NBC and its substitutes possess anticonvulsant activity [20]. Compared with BCA, NBC showed a stronger antipyretic effect [21]. Additionally, among NBC, BCS, and BCA, BCA is the most widely used component in Chinese patent medicines [16]. However, there is a lack of systematic evidence on whether these substitutes can serve as effective alternatives to NBC in exerting anti-seizure effects.

Various animal models of epilepsy have been established to mimic different seizure types [22]. Among them, the pentylenetetrazol (PTZ)-induced model is a widely used pharmacological tool for acute seizure induction [23] and is frequently employed to evaluate the efficacy of anti-seizure medications [24]. Therefore, in this study, we evaluated the anticonvulsant efficacy of NBC, BCS and BCA using a PTZ-induced acute seizure mouse model. By integrating behavioral assessments, histopathological examinations, neurotransmitter measurements, and inflammatory analyses, we systematically compared their pharmacological characteristics and substitution potential. Network pharmacology combined with molecular docking was used as a complementary approach to predict potential signaling pathways. The findings provide experimental evidence for the comparative evaluation of different Bovis Calculus sources and contribute to the rational development and application of alternative Bovis Calculus resources.

## 2. Materials and Methods

### 2.1. Animals

Male ICR mice weighing 20 ± 2 g were obtained from the Experimental Animal Center of Ningxia Medical University (license: SCXK(Ning) 2020-0001) and housed under specific pathogen-free (SPF) conditions. The temperature of the animal chamber was maintained at 23 ± 2 °C with 12 h of daylight and 12 h of darkness. All animal procedures were approved by the Institutional Ethics Committee of Ningxia Medical University (Approval No. IACUC-NYLAC-2023-238).

### 2.2. Drugs and Reagents

BCA, NBC, and BCS were obtained from Sichuan Co-creation Pharmaceutical Co., Ltd. (Pengzhou, China), Bozhou Chinese Herbal Medicine Market (Bozhou, China), and Wuhan Jianmin Dapeng Pharmaceutical Co., Ltd. (Wuhan, China), respectively. Prior to administration, each substance was suspended in a 0.5% sodium carboxymethyl cellulose (CMC-Na) solution. Sodium Valproate (VPA) was purchased from Hunan Xiangzhong Pharmaceutical Co., Ltd. (1H240510, Shaoyang, China). PTZ was purchased from Shanghai Aladdin Biochemical Technology Co., Ltd. (G2305131, Shanghai, China).

### 2.3. Experimental Design

In the study of the anti-seizure effects of BCA, the mice were divided into six groups of twelve mice each: the control group, the model group, the positive group, the BCA-L group, the BCA-M group, and the BCA-H group. According to a previous report, the effective dose of Bovis Calculus against seizures in mice is 600 mg/kg [20]. Therefore, three doses of BCA (400, 600, and 800 mg/kg) were selected for this study to verify its anti-seizure activity and to identify its effective dose range. The mice in the control group and model group were intragastrically administered a vehicle solution consisting of 0.5% CMC-Na for 10 days. The mice in the positive group were intragastrically administered VPA at a dose of 250 mg/kg for 10 days. The mice in the BCA-L group, BCA-M group, and BCA-H group were intragastrically administered BCA at a dose of 400 mg/kg, 600 mg/kg, and 800 mg/kg, respectively, for 10 days. After 1 h, when the last administrated drugs, except for the control group, the other groups were injection of PTZ (70 mg/kg) [25].

In the study of the anti-seizure effects of NBC and its substitutes, the mice were divided into six groups, including the control group, the model group, the positive group, the NBC group, the BCS group, and the BCA group. Based on the results of the BCA anti-seizure study, a dose of 600 mg/kg was selected. All other experimental procedures were identical to those described as BCA with different doses.

### 2.4. Seizure Observation

Following injection, the mice were closely observed for a period of 30 min. Seizure behavior was observed. The intensity was scored according to Racine’s score as follows: Stage 0, no response (no convulsions); Stage I, facial tremors; Stage II, generalized wave-like spasms; Stage III, unilateral forelimb clonus; Stage IV, bilateral forelimb clonus and rearing; Stage V, collapse with generalized seizure [26]. Meanwhile, the seizure latency and seizure duration were recorded.

### 2.5. Histological Analysis

Hematoxylin and Eosin (H&E) staining and Nissl staining were conducted to assess neuronal damage within the hippocampal CA1, CA3 and DG regions of control and PTZ-induced seizure mouse models. After seizure observation, the brains were harvested and placed in a 4% paraformaldehyde solution for overnight fixation. Then, it was embedded in paraffin and sliced into 5 μm thick sections by a microtome.

For H&E staining, slices were deparaffinized using xylene and rehydrated by graded concentrations of alcohol. Then, it was stained in hematoxylin for 5 min to label nuclei. Following washing with water and differentiation in 1% acid alcohol, the slices were counterstained in eosin staining solution for 2 min to visualize the cytoplasm and other tissue components. The stained slices were then dehydrated with graded alcohol and mounted using a coverslip. A digital pathology scanner and analysis system (Aperio LV1, Vista, CA, USA) was applied to examine H&E-stained slices. Histopathologic scores were determined based on the percentage of damaged neurons using the following semiquantitative analysis: normal, no injury or rare isolated apoptotic neuron = 0, rare neuronal injury (<5 clusters) = 1, occasional neuronal injury (5–15 clusters) = 2, frequent neuronal injury (>15 clusters) = 3, diffuse neuronal injury = 4 [27].

Nissl staining was used to label Nissl bodies in neurons [28]. The slices were deparaffinized and rehydrated following a procedure similar to that used for H&E staining. Subsequently, they were stained in a 0.1% cresyl violet solution for 10 min, followed by rinsing with water, differentiation in 95% alcohol, dehydration through absolute alcohol, and coverslipping. A digital pathology scanner and analysis system (Aperio LV1, Leica Biosystems Imaging, Inc., Vista, CA, USA) was used to examine the Nissl staining slices. The number of neurons was counted using ImageJ 1.49p.

### 2.6. Biochemical Analyses with ELISA

Hippocampal tissue was homogenized in cold phosphate-buffered saline and centrifuged at 10,000× *g* for 15 min at 4 °C. The supernatant was collected to determine the concentration of neurotransmitters, including serotonin (5-HT), γ-Aminobutyricacid (GABA), and dopamine (DA), as well as inflammatory factors (IL-6, IL-1β, and TNF-α). ELISA kits from COIBO BIO (Shanghai Coibo Biotechnology Co., Ltd., Shanghai, China) were used according to the manufacturer’s instructions. The absorbance was measured by a microplate reader at the recommended wavelengths, and the concentrations were calculated based on the standard curves.

### 2.7. Network Pharmacology

In this study, the network pharmacology methodology was based on the method reported by Men et al. with minor modifications [29]. Our previous studies have identified 19 major compounds in BCA samples (Table 1). The chemical structures of the compounds were drawn using ChemBioDraw Ultra 14.0 and converted to MDL SD files. Corresponding SMILES strings were obtained from the PubChem database (https://www.ncbi.nlm.nih.gov/, accessed on 5 December 2025). Target predictions were performed by uploading the MDL SD file of the 19 major compounds in BCA to the PharmMapper database (https://www.lilab-ecust.cn/pharmmapper/, accessed on 15 December 2025) and submitting the SMILES strings to the SwissTargetPrediction database. Disease-related targets were obtained by searching the keyword “seizure” or “epilepsy” in the GeneCard database (https://www.genecards.org/, accessed on 25 December 2025), Therapeutic Target Database (TTD, https://ttd.idrblab.cn/, accessed on 25 December 2025), and PharmGKB databases (https://www.clinpgx.org/, accessed on 25 December 2025). The predicted targets of the compounds and disease-related targets were merged and deduplicated, and the common targets were identified using the Venny online tool (https://bioinfogp.cnb.csic.es/tools/venny/index.html, accessed on 29 December 2025). Then, the common targets were imported into the STRING database (https://cn.string-db.org/, accessed on 29 December 2025) to construct a drug–component–target–disease network and protein–protein interaction (PPI) networks. Cytoscape 3.10.2 was used to visualize the network and to analyze topological parameters, including betweenness centrality, closeness centrality and degree centrality. Nodes with values greater than the median were considered key targets. These targets were imported into the DAVID database (https://davidbioinformatics.nih.gov/, accessed on 30 December 2025) for functional enrichment analysis. Homo sapiens was selected as the species. The top ten Gene Ontology terms and the top twenty pathways, ranked by *p*-values, were visualized.

### 2.8. Molecular Docking

Taurine and bilirubin were identified as the main active compounds. Based on the results of the drug–component–target–disease network analysis, key targets with high topological parameters were selected for docking studies. The three-dimensional structures of the selected targets were downloaded from the PDB database (https://www.rcsb.org/, accessed on 2 January 2026). Protein preparation included the removal of water molecules and co-crystallized ligands, followed by the addition of hydrogen atoms and energy minimization. Molecular docking was performed using Discovery Studio 2019, and the binding affinity was evaluated by docking scores.

### 2.9. Statistical Analysis

GraphPad Prism 7 Software (GraphPad Software, San Diego, CA, USA) was used for statistical data analysis. The data are expressed as mean ± standard error of the mean (SEM). For the results of histological analysis and biochemical analyses with ELISA, a one-way ANOVA followed by Dunnett’s test was used for comparisons of multiple groups against the control group. Survival data were analyzed using the Kaplan–Meier method and compared among multiple groups (*n* = 10 per group) using the log-rank (Mantel–Cox) test. A *p*-value of less than 0.05 was considered statistically significant for all tests.

## 3. Results

### 3.1. BCA Exerts Dose-Dependent Protective Effects Against PTZ-Induced Acute Seizures Through Neuroprotection and Modulation of Neurotransmitters and Neuroinflammation

#### 3.1.1. BCA Alleviates PTZ-Induced Acute Seizure Behaviors

**Figure 1:**
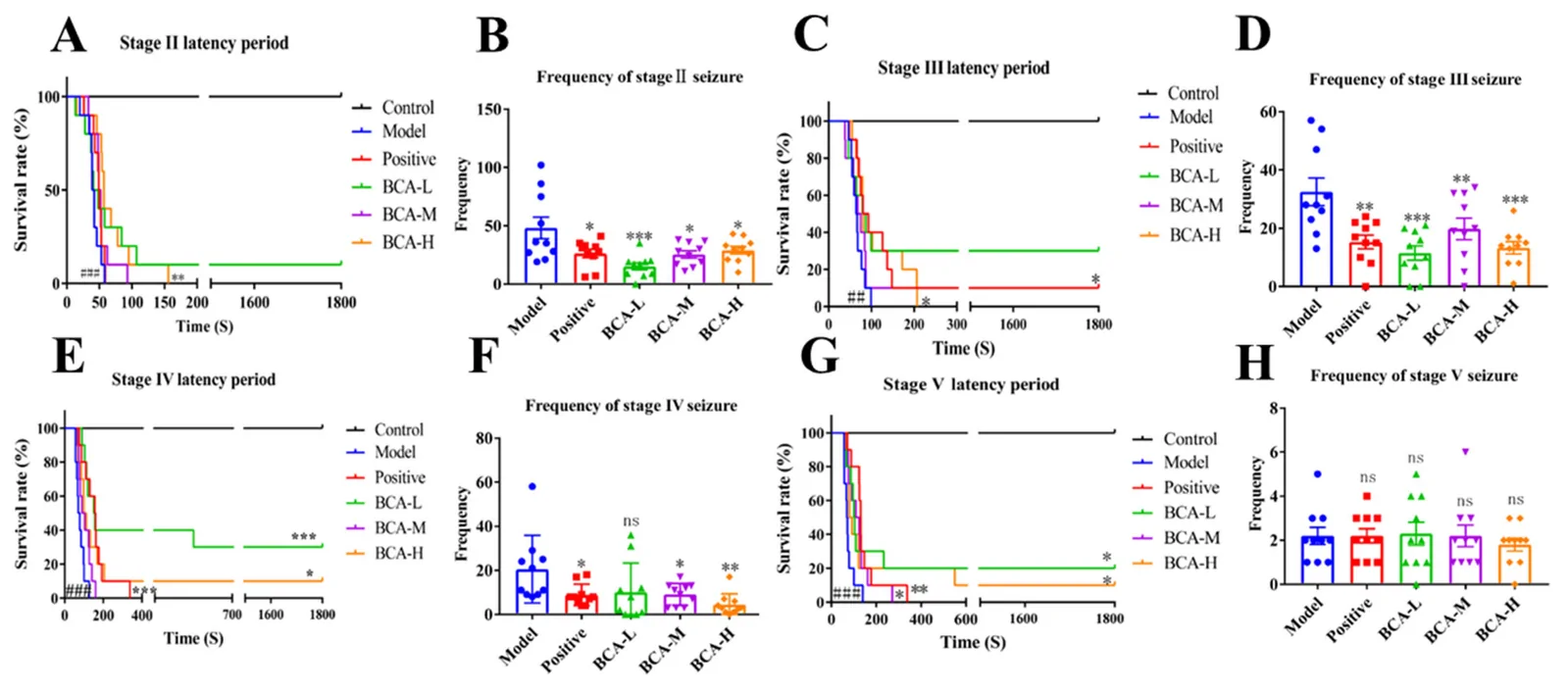
Bovis Calculus Artifactus (BCA) exhibits anti-seizure effect in a PTZ-induced acute seizure model. (**A**–**D**) Survival curve of mice based on the latency period of stage II seizures to stage V seizures. (**E**–**H**) The frequency of stage II seizures to stage V seizures. Compared with the control group, ## *p* < 0.01, ### *p* < 0.001; compared with the model group, * *p* < 0.05, ** *p* < 0.01, *** *p* < 0.001; ns, not significant.

The effect of BCA on seizures was evaluated in PTZ-induced seizure mouse models by monitoring the latency period to seizure onset and the frequency of seizures across different stages (stage II to stage V). A survival curve was generated to analyze the latency period to seizure onset from stage II to stage V (Figure 1A–D). No seizures were observed in the control group. In contrast, the model group was significantly different (*p* < 0.001), confirming the successful induction of acute seizures by PTZ. A significant difference in survival was observed among the groups (*p* < 0.001), which was most apparent in the early phase. BCA demonstrated a clear trend of dose-dependent effect. Compared with the model group, the BCA-H group exhibited a significant prolongation of latency to seizure onset from stage II to stage V (*p* < 0.05 or *p* < 0.01, Figure 1A–D). Meanwhile, treatment with BCA at all doses significantly reduced the frequency of stage II and stage III seizures (*p* < 0.05 or *p* < 0.01, Figure 1E,F). Furthermore, the medium and high doses of BCA (BCA-M and BCA-H) also significantly reduced the frequency of stage IV seizures (*p* < 0.05 and *p* < 0.01, Figure 1G). Taken together, these findings demonstrate that BCA exerts protective effects against PTZ-induced acute seizures.

#### 3.1.2. BCA Protects Against PTZ-Induced Neuronal Damage

**Figure 2:**
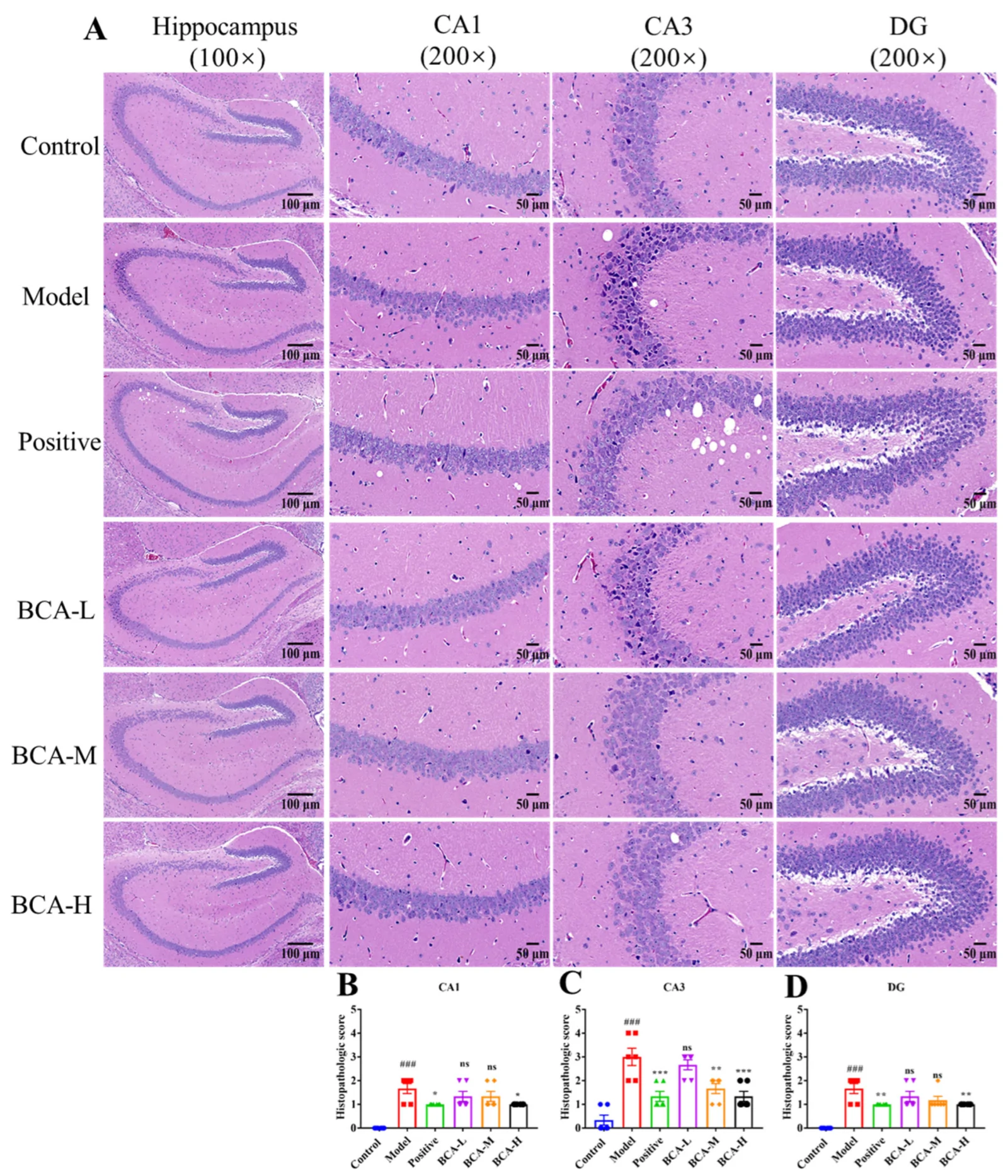
BCA exhibits neuroprotective effect in a PTZ-induced acute seizure model, as assessed by H&E staining. (**A**) Representative H&E staining images of neuronal damage in the hippocampus. The histopathologic scores of CA1 (**B**), CA3 (**C**), and DG (**D**) regions. Compared with the control group, ### *p* < 0.001; compared with the model group, * *p* < 0.05, ** *p* < 0.01, *** *p* < 0.001; ns, not significant.

To evaluate the neuroprotective potential of BCA, neuronal damage in the hippocampus was first assessed by H&E staining (Figure 2). Compared with the control group, the model group exhibited loosely arranged hippocampal neurons with more deeply stained nuclei, particularly in the CA3 region. BCA treatment alleviated these pathological changes, resulting in neurons that were more closely packed (Figure 2A). Histopathologic scores based on semiquantitative analysis demonstrated that BCA treatment alleviated PTZ-induced hippocampal neuronal damage, which was more pronounced in the CA3 region at the higher dose (Figure 2B–D).

**Figure 3:**
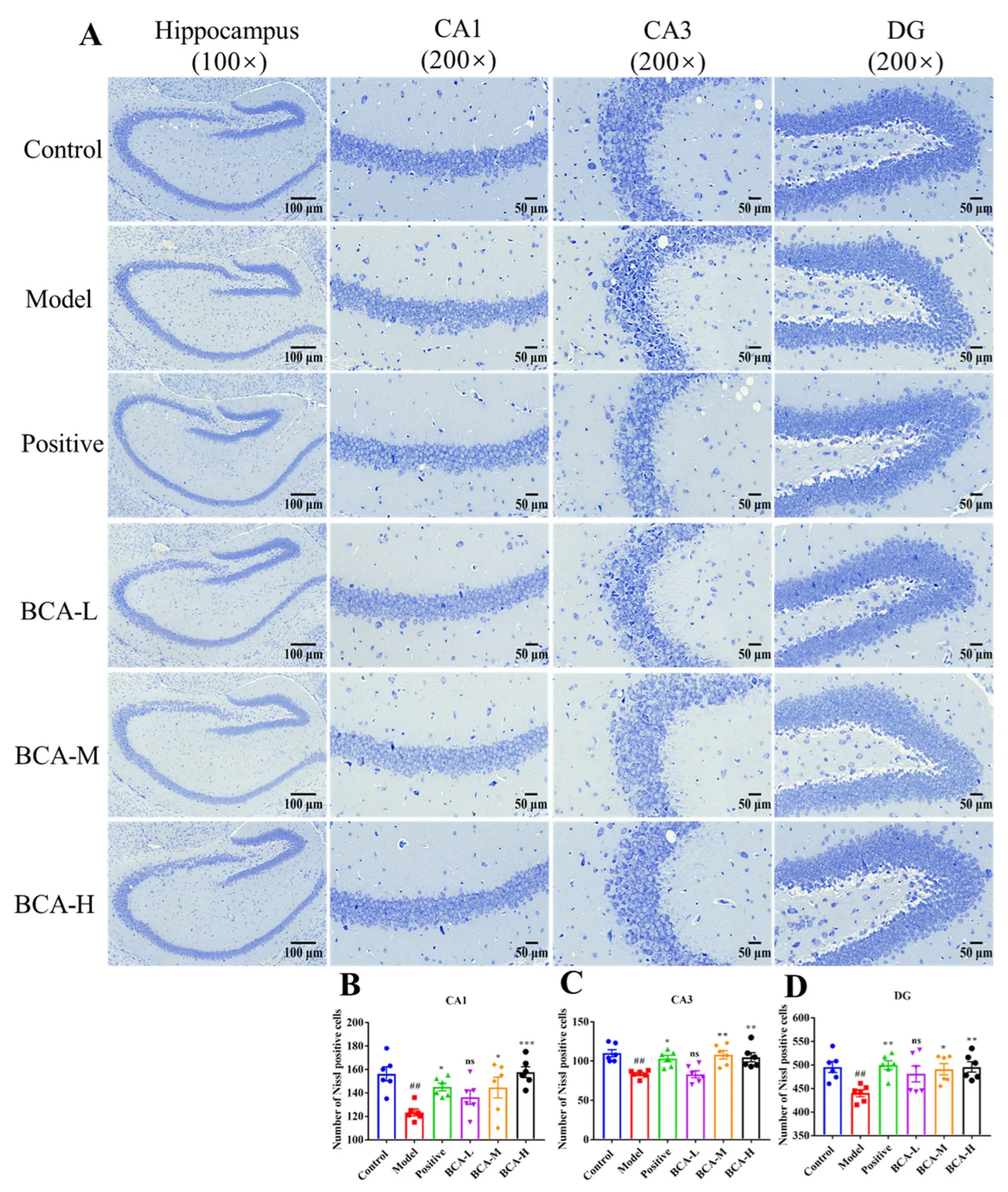
BCA exhibits a neuroprotective effect in a PTZ-induced acute seizure model, as assessed by Nissl staining. (**A**) Representative Nissl staining images of neuronal damage and cell viability in the hippocampus. The number of Nissl-positive cells in CA1 (**B**), CA3 (**C**), and DG (**D**) regions of the hippocampus. Compared with the control group, ## *p* < 0.01; compared with the model group, * *p* < 0.05, ** *p* < 0.01, *** *p* < 0.001; ns, not significant.

Furthermore, Nissl staining was performed to quantitatively assess neuronal damage and cell viability (Figure 3A–D). Compared with the control group, the model group showed a significant reduction in the density of Nissl-positive cells (*p* < 0.01, Figure 3B–D). BCA treatment significantly restored neuronal counts in the CA1 (*p* < 0.05 or *p* < 0.001, Figure 3B), CA3 (*p* < 0.01, Figure 3C) and DG (*p* < 0.05 or *p* < 0.01, Figure 3D) regions of the BCA-M and BCA-H groups, demonstrating mitigation of seizure-related neuronal loss. This protective effect was dose-dependent. These findings demonstrate the neuroprotective activity of BCA in the PTZ-induced acute seizure model.

#### 3.1.3. BCA Ameliorates the PTZ-Induced Abnormal Alterations in the Levels of Neurotransmitters and Inflammatory Cytokines

**Figure 4:**
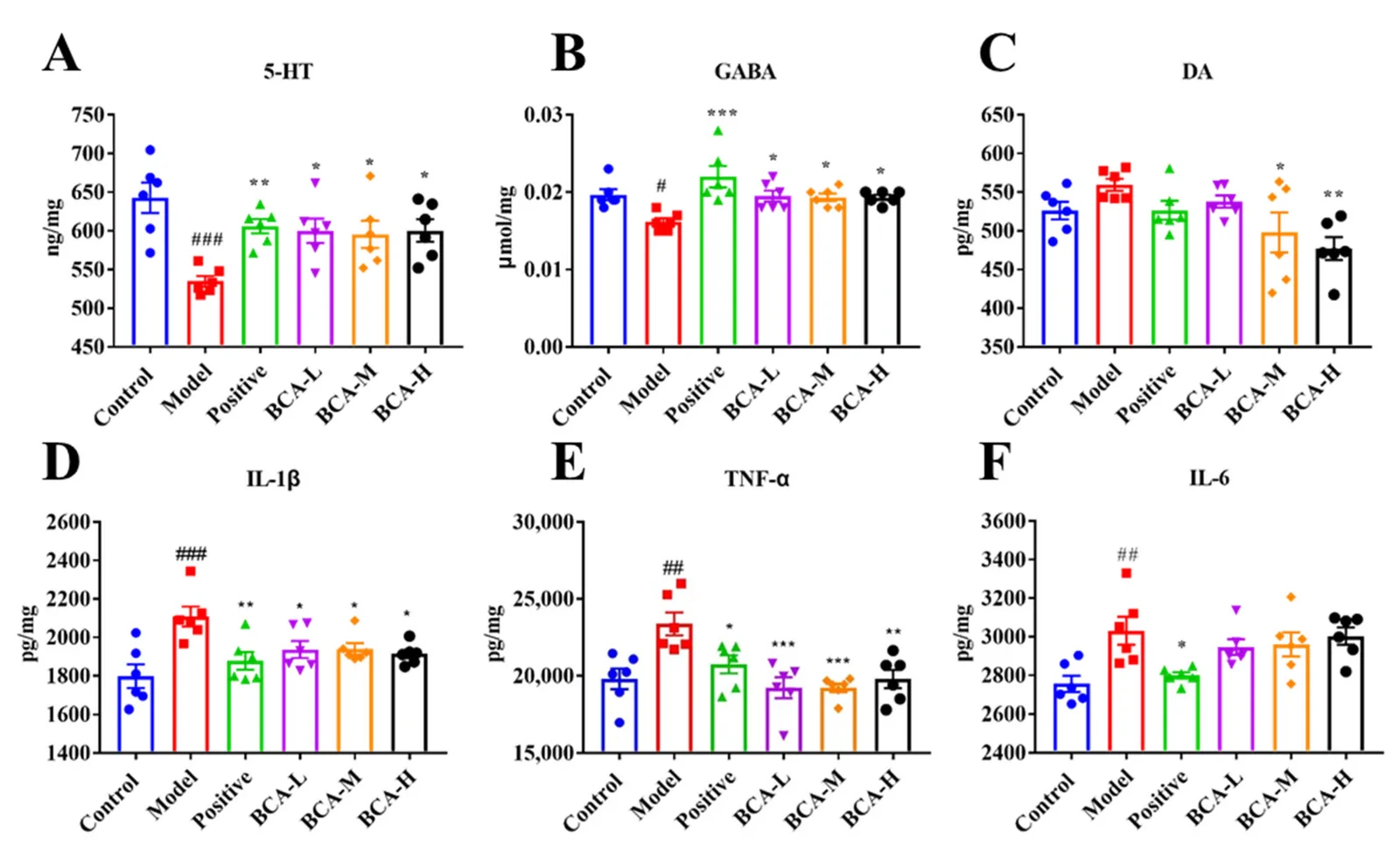
BCA modulates neurotransmitters (**A**–**C**) and inhibits neuroinflammatory factors (**D**–**F**). Compared with the control group, # *p* < 0.05, ## *p* < 0.01, ### *p* < 0.001; compared with the model group, * *p* < 0.05, ** *p* < 0.01, *** *p* < 0.001.

Seizures are associated with abnormal neurotransmitter kinetics [30]. Therefore, the expression levels of 5-HT, GABA and DA were measured to investigate the potential mechanism behind the anti-seizure action of BCA in a PTZ-induced acute mouse seizure model. Both 5-HT and GABA levels in the hippocampus were significantly decreased in the model group compared with the control group (*p* < 0.001 or *p* < 0.05). In contrast, BCA treatment significantly reversed this decline (*p* < 0.05, Figure 4A,B). DA content increased in the model group compared with the control group, but no significant difference was observed (Figure 4C). It is worth noting that BCA treatment decreased the content of DA, especially at medium and high doses, which showed a significant effect (*p* < 0.05 or *p* < 0.01, Figure 4C). Taken together, the results suggest that BCA exhibited protective effects against PTZ-induced acute seizures by modulating the levels of multiple neurotransmitters.

Neuroinflammation is also an important factor leading to seizures. To investigate this, ELISA was performed to quantify the concentration of IL-1β, IL-6 and TNF-α. The expression levels of IL-1β (Figure 4D), TNF-α (Figure 4E) and IL-6 (Figure 4F) were significantly increased in PTZ-induced seizure models compared with the control group (*p* < 0.001, *p* < 0.01, *p* < 0.01). BCA pre-treatment significantly reduced the levels of IL-1β (*p* < 0.05) and TNF-α (*p* < 0.01). In contrast, IL-6 expression was not significantly altered by BCA treatment (Figure 4F). Collectively, these findings indicate that the protective effects against PTZ-induced acute seizures of BCA are mediated, at least in part, by the suppression of specific pro-inflammatory cytokines.

### 3.2. Comparative Efficacy of NBC and Its Substitutes Against PTZ-Induced Acute Seizures

The above results indicated that BCA can dose-dependently reduce the frequency and duration of seizure activity. Its protective effects against PTZ-induced acute seizures may involve attenuation of neuronal injury, modulation of neurotransmitter levels, and inhibition of neuroinflammatory responses. Since the mid-dose (600 mg/kg) already exhibited marked protective effects, it was selected for subsequent comparative assessment of the protective effects of NBC and its substitutes against PTZ-induced acute seizures.

#### 3.2.1. Behavioral Assessment

**Figure 5:**
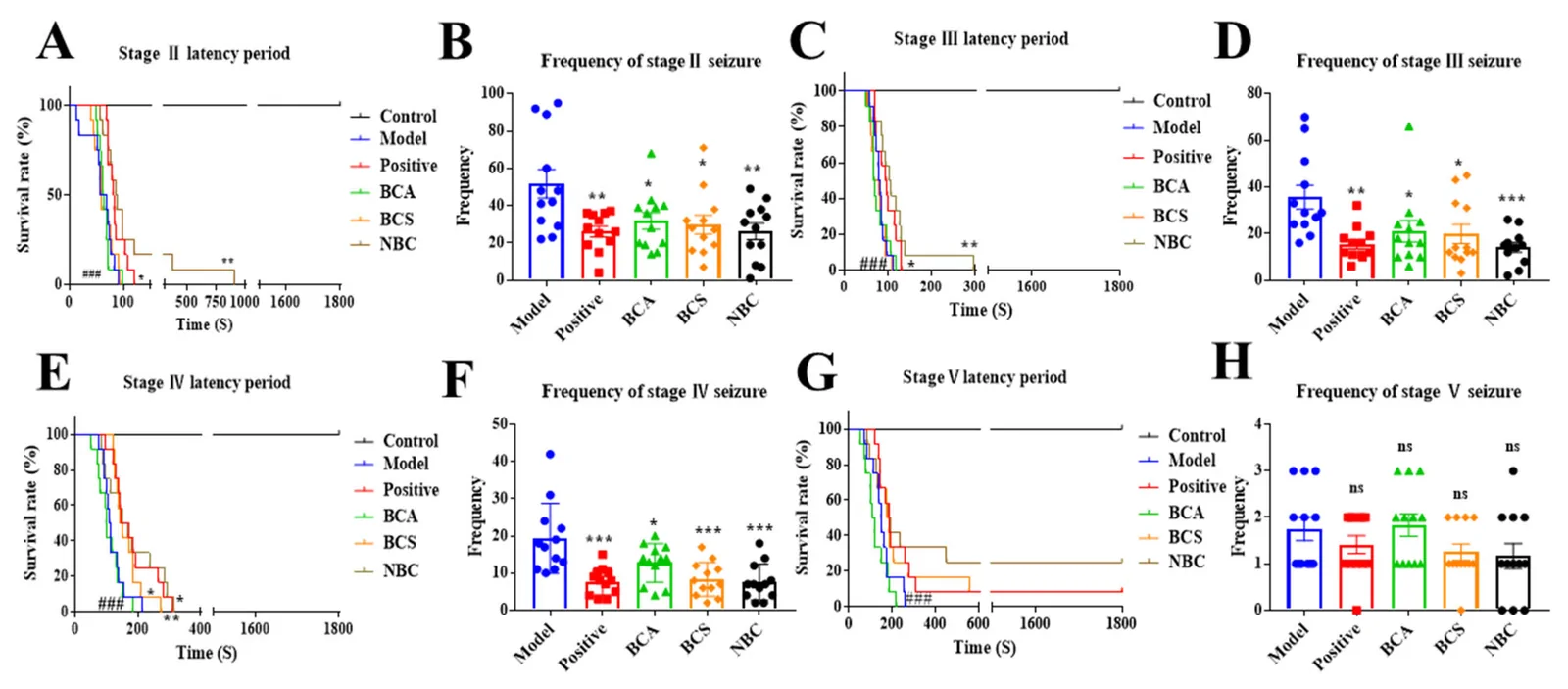
Behavioral assessment of Bovis Calculus (NBC) and its substitutes. (**A**–**D**) Survival curve of mice based on the latency period of stage II seizures to stage V seizures. (**E**–**H**) The frequency of stage II seizures to stage V seizures. Compared with the control group, ### *p* < 0.001; compared with the model group, * *p* < 0.05, ** *p* < 0.01, *** *p* < 0.001; ns, not significant.

Survival curve analysis revealed that seizure latency was significantly shortened in the model group compared with the control group (*p* < 0.001), confirming the successful establishment of the acute PTZ-induced seizure model (Figure 5A–D). In mice pretreated with NBC, it significantly prolonged the seizure latency of stages II, III, and IV, and mortality risk was reduced (*p* < 0.05 or *p* < 0.01, Figure 5A–C). BCA showed no significant effects on seizure latency across all stages (*p* > 0.05), whereas BCS significantly prolonged the latency of stage IV seizures only (*p* < 0.05).

Analysis of seizure frequency demonstrated that all tested preparations of NBC and its substitutes significantly reduce the number of stage II, III, and IV seizure events (*p* < 0.05, *p* < 0.01 or *p* < 0.001, Figure 5E–H).

Overall, these results suggest that NBC and its substitutes all exhibit protective effects against PTZ-induced acute seizures, but their effects on seizure latency were notably divergent. Among them, NBC exhibited the broadest spectrum of action by prolonging seizure latency across multiple stages; BCS showed a limited effect, extending latency only for stage IV seizures; whereas BCA had no significant impact on seizure latency, suggesting that their protective effects may be primarily attributable to the reduction in seizure frequency.

#### 3.2.2. Neuroprotective Effect Assessment

**Figure 6:**
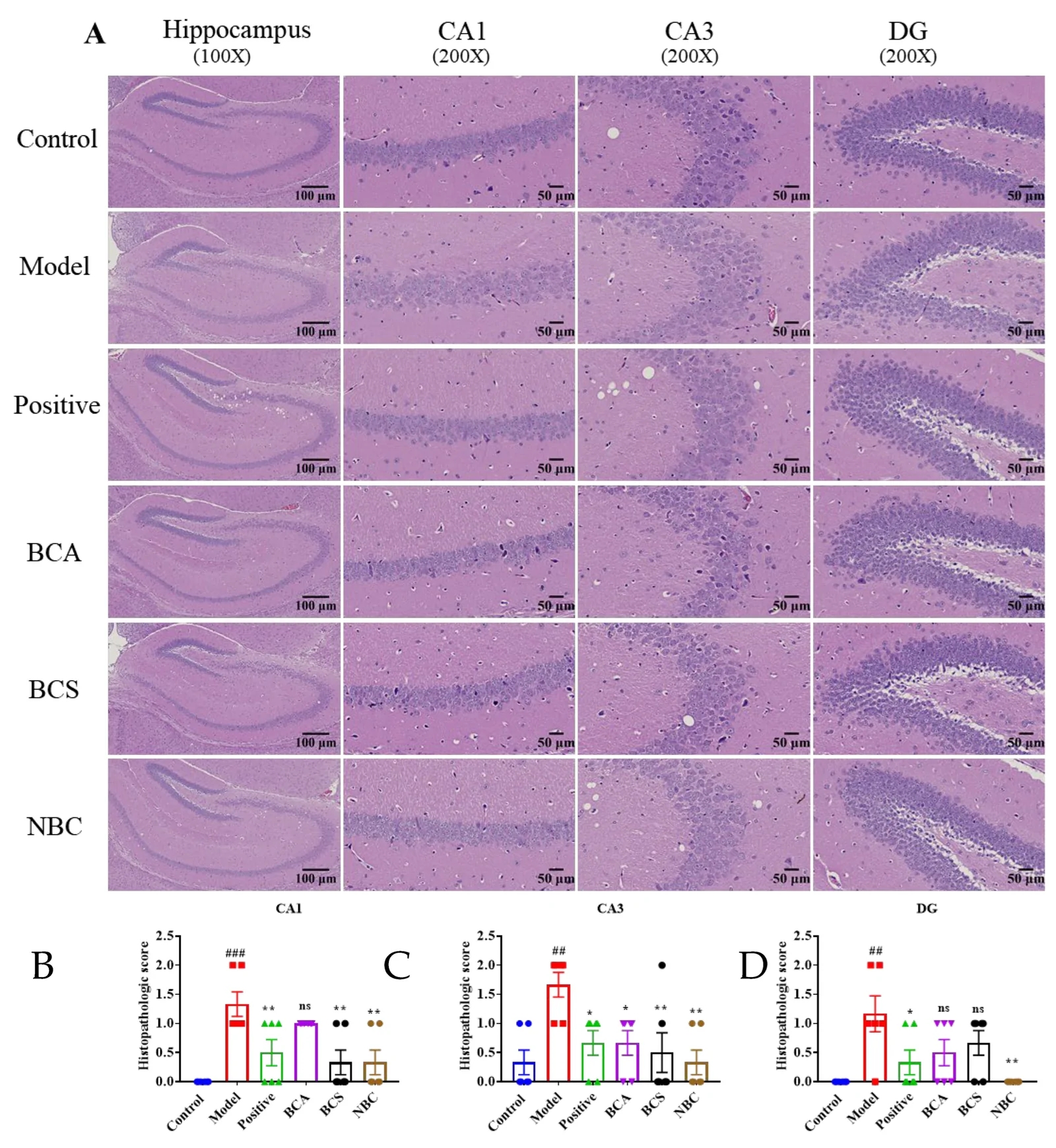
Neuroprotective effect assessment of NBC and its substitutes by H&E staining. (**A**) Representative H&E staining images of neuronal damage in the hippocampus. The histopathologic scores of CA1 (**B**), CA3 (**C**), and DG (**D**) regions. Compared with the control group, ## *p* < 0.01, ### *p* < 0.001; compared with the model group, * *p* < 0.05, ** *p* < 0.01; ns, not significant.

H&E staining results (Figure 6) showed that, compared with the control group, the model group exhibited loosely arranged neurons, reduced neuronal numbers, and elevated histopathological scores in the CA1, CA3 and DG regions of the hippocampus. Preventive administration of NBC and its substitutes improved neuronal arrangement to varying extents. Among the treatment groups, NBC produced the lowest histopathological scores across all three regions. The BCS group showed improvement in the CA1 and CA3 regions. The BCA group exhibited a lower score only in the CA3 region relative to the model group.

**Figure 7:**
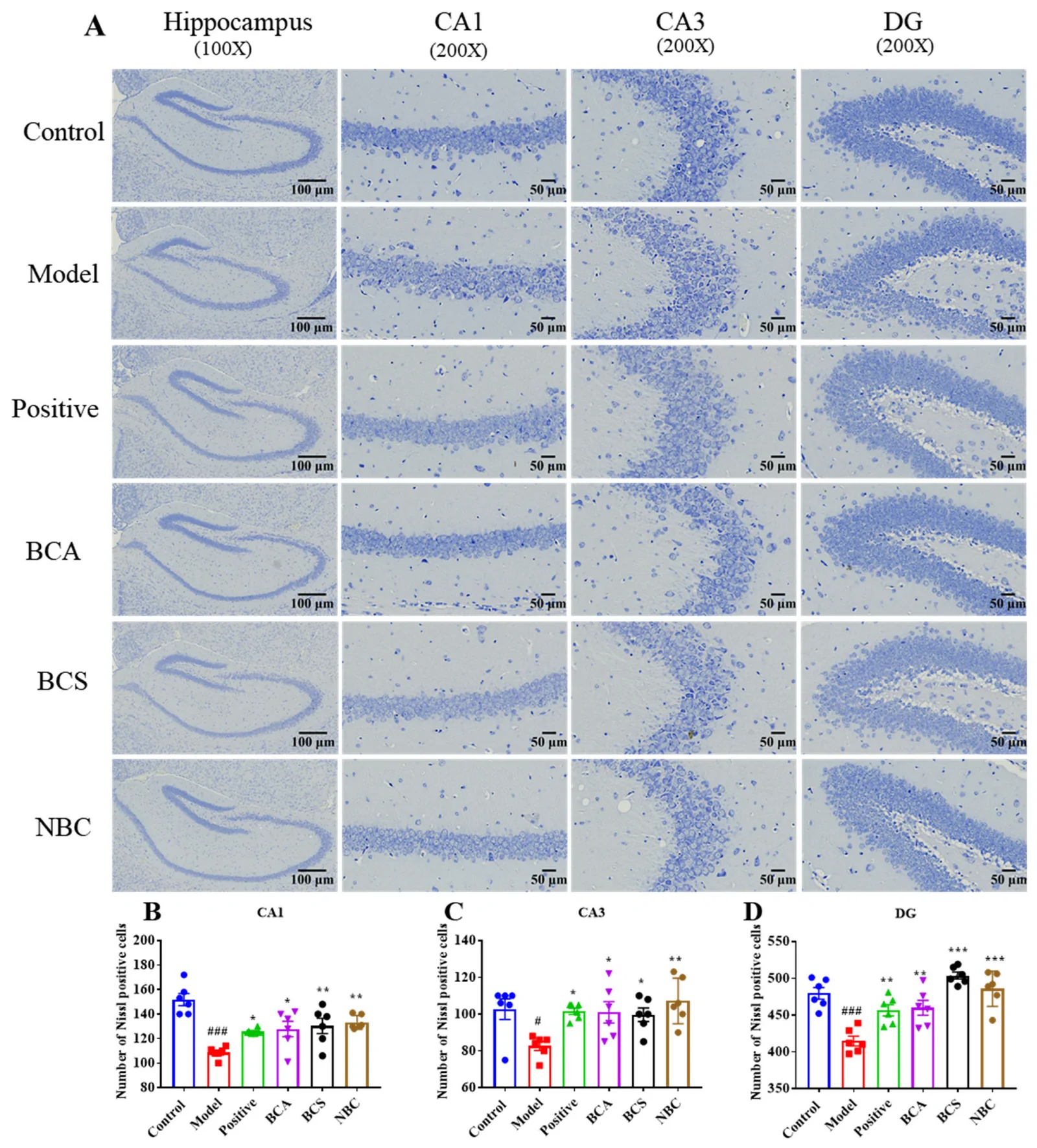
Neuroprotective effect assessment of NBC and its substitutes by Nissl staining. (**A**) Representative Nissl staining images of neuronal damage and cell viability in the hippocampus. The number of Nissl-positive cells in CA1 (**B**), CA3 (**C**), and DG (**D**) regions of the hippocampus. Compared with the control group, # *p* < 0.05, ### *p* < 0.001; compared with the model group, * *p* < 0.05, ** *p* < 0.01, *** *p* < 0.001.

Nissl staining results (Figure 7) revealed significantly decreased neuronal counts in all hippocampal subregions of the model group (*p* < 0.01). Following preventive treatment, neuronal counts in all three regions were significantly higher in the BCA, BCS and NBC groups than in the model group (*p* < 0.05, *p* < 0.01, or *p* < 0.001).

The two staining methods consistently revealed significant damage across all hippocampal subregions in the model group relative to the control group, confirming successful establishment of the acute seizure model. All tested agents exerted certain neuroprotective effects following preventive intervention, though with region-selective profiles. NBC provided the most comprehensive morphological protection, as evidenced by improvement in all three regions on H&E staining. BCA, BCS and NBC demonstrated prominent pro-neuronal survival effects, with efficacy in all three regions on Nissl staining. Overall, these findings suggest that the neuroprotective effects of the tested agents display distinct regional selectivity.

#### 3.2.3. Neurotransmitter and Inflammatory Cytokines Assessment

**Figure 8:**
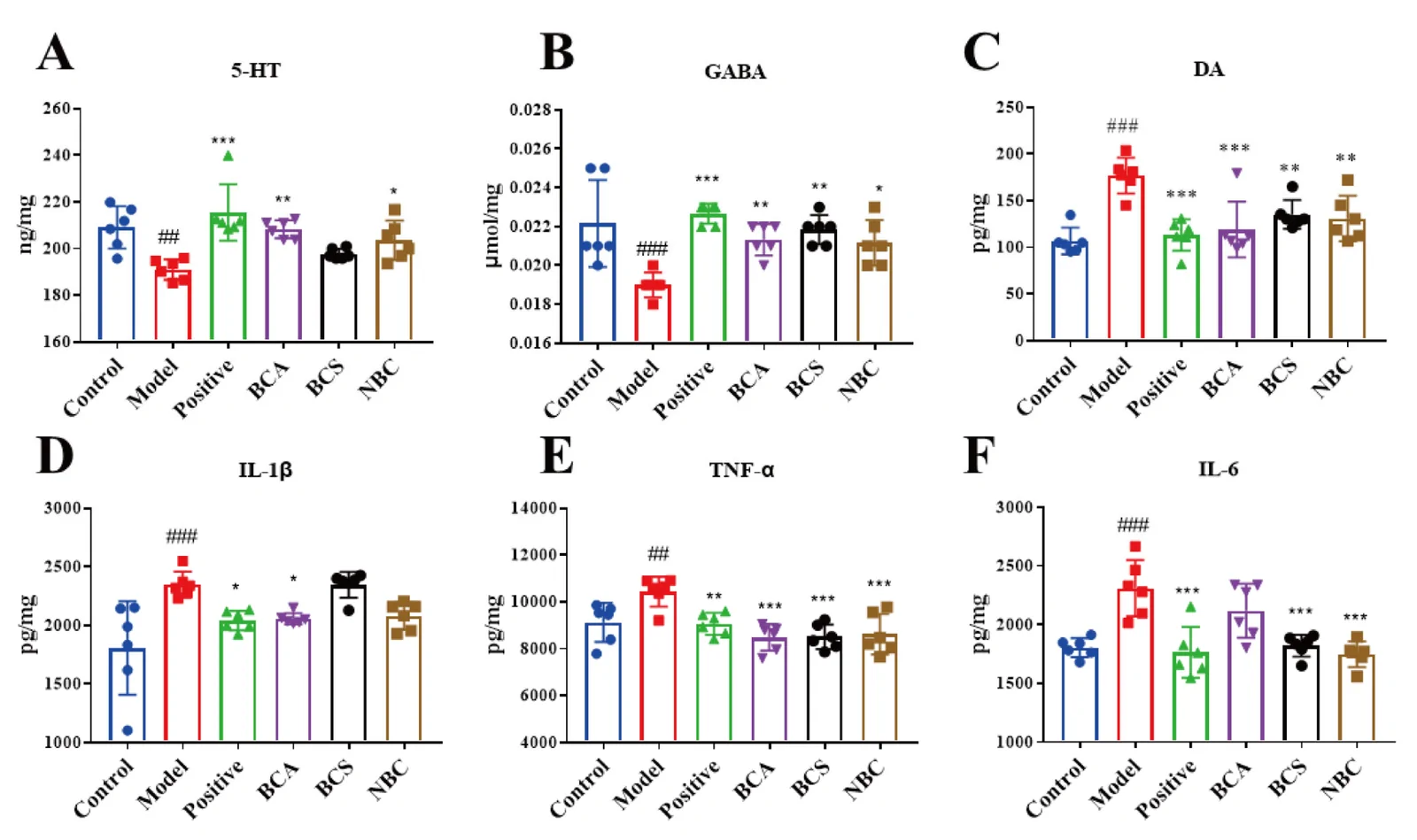
Neurotransmitter (**A**–**C**) and inflammatory cytokines (**D**–**F**) assessment of NBC and its substitutes. Compared with the control group, ## *p* < 0.01, ### *p* < 0.001; compared with the model group, * *p* < 0.05, ** *p* < 0.01, *** *p* < 0.001.

Neurotransmitter assays in hippocampal tissues revealed that, compared with the control group, the model group exhibited significantly decreased GABA and 5-HT levels (*p* < 0.01, Figure 8A,B), and significantly increased DA levels (*p* < 0.01, Figure 8C). Preventive administration of NBC and its substitutes reversed these changes to varying degrees. The BCA and NBC groups showed significant improvements in all three neurotransmitters (GABA, 5-HT and DA). The BCS group showed significant regulation of GABA and DA (*p* < 0.001, *p* < 0.01), but its effect on 5-HT did not reach statistical significance (*p* > 0.05).

Inflammatory cytokine assays (Figure 8D–F) showed that, compared with the control group, the model group had significantly elevated IL-1β, IL-6 and TNF-α levels in the hippocampus (*p* < 0.001, *p* < 0.05, *p* < 0.001). Following preventive treatment, all drug groups exhibited significant downregulation of TNF-α (*p* < 0.01 or *p* < 0.001). IL-1β levels showed a decreasing trend in all treatment groups, but only the BCA group reached statistical significance (*p* < 0.05). For IL-6, all groups showed a decreasing trend, with significant reductions observed in the BCS and NBC groups, whereas the BCA group did not reach statistical significance.

Integrating the neurotransmitter and cytokine data, the model group displayed clear neurotransmitter imbalance (reduced GABA/5-HT, elevated DA) and inflammatory activation (elevated IL-1β, IL-6, TNF-α) in the hippocampus. Preventive treatment with NBC and its substitutes corrected these abnormalities to different extents, though with distinct regulatory profiles. The BCA group exhibited prominent effects on neurotransmitter regulation (GABA, 5-HT, DA) and IL-1β suppression, but showed limited efficacy against IL-6. The NBC groups showed broad regulation of neurotransmitters, but had limited suppressive effects on IL-1β. The BCS group showed relatively restricted neurotransmitter modulation (ineffective on 5-HT), yet exerted significant suppression of IL-6 and TNF-α. These findings suggest that the anti-seizure effects of NBC and its substitutes may be closely associated with the restoration of neurotransmitter balance and the inhibition of neuroinflammation in the hippocampus, though the regulatory targets and potencies vary among the substitutes.

### 3.3. NBC and Its Substitutes Anti-Seizure Target Identification

#### 3.3.1. Screening of Major Compounds in NBC and Its Substitutes and Seizure-Related Targets

**Figure 9:**
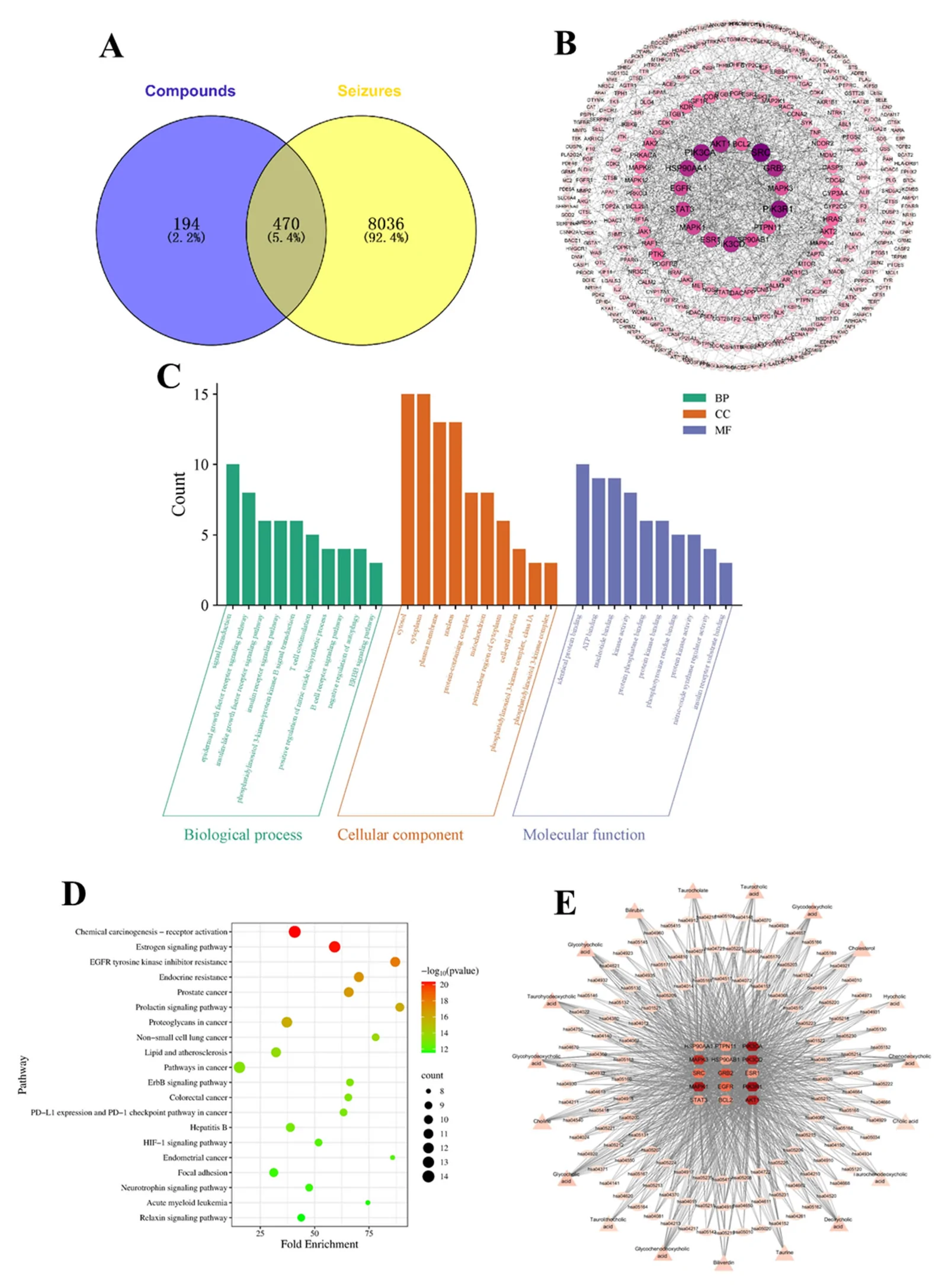
Network pharmacology analysis. (**A**) Intersection target analysis. (**B**) PPI network. (**C**) GO enrichment analysis. (**D**) KEGG enrichment analysis. (**E**) Drug–component–target–disease network.

A total of 664 component targets were obtained from the PharmMapper and the SwissTargetPrediction databases, while 8506 seizure-related disease targets were collected from the GeneCard, TTD, and PharmGKB databases. Among them, 470 overlapping targets were shared between the component targets and the seizure-related disease targets (Figure 9A).

#### 3.3.2. PPI Network Analysis

To identify the key targets and explore the relationship between target interactions, a PPI network was constructed (Figure 9B). This network consists of 335 interacting targets and 1208 interacting edges, highlighting the complexity and interconnectivity of the targets. Among these, 15 targets were identified as key proteins based on their centrality in the network, including Proto-oncogene tyrosine-protein kinase Src (SRC), Phosphatidylinositol 3-kinase regulatory subunit alpha (PIK3R1), Phosphatidylinositol 4,5-bisphosphate 3-kinase catalytic subunit alpha isoform (PIK3CA), RAC-alpha serine/threonine-protein kinase (AKT1), and GRB2-associated-binding protein 2 (GRB2).

#### 3.3.3. GO and KEGG Analyses

GO enrichment analysis and KEGG analysis were performed to explore the underlying mechanisms of NBC and its role in seizures. The first ten terms of GO enrichment analysis are shown in Figure 9C. Fifteen key targets were involved in 171 GO Biological Processes (BP), including signal transduction, epidermal growth factor receptor signaling pathway, insulin-like growth factor receptor signaling pathway, insulin receptor signaling pathway, phosphatidylinositol 3-kinase/protein kinase B signal transduction. These multiple signal pathways may contribute to neuroprotection, cell proliferation, anti-apoptosis effects, and anti-inflammation effects [31,32]. In the Cellular Component (CC) category (Figure 9C), fifteen key targets were mainly enriched in the cytoplasm and plasma membrane, indicating that they are primarily distributed in the intracellular fluid and at the cell surface, which is consistent with their roles in signal reception and transduction. In the Molecular Function (MF) category, fifteen key targets mainly regulated identical protein binding, ATP binding, nucleotide binding, and kinase activity, suggesting that they are involved in protein–protein interactions, enzymatic activity, and signal transduction processes. KEGG pathway analysis revealed 133 pathways, and the first 20 pathways are shown in Figure 9D, which include chemical carcinogenesis-receptor activation, estrogen signaling pathway, pathways in cancer, proteoglycans in cancer, and others. It has been reported that chemical carcinogenesis and signaling pathways in cancer are associated with inflammatory response [33,34], while estrogen signaling integrity is associated with neuroprotective effects in neurodegeneration [35]. The above results suggested that NBC and its substitutes exert synergistic “multicomponent–multitarget–multipathway” regulatory effects on seizures.

#### 3.3.4. Drug–Component–Target–Disease Network

To illustrate the interactions among drugs, active compounds, targets, and disease, a drug–component–target–disease network was constructed based on the fifteen key targets (Figure 9E). Network analysis identified nine key targets according to betweenness centrality, closeness centrality and degree centrality, including PIK3R1, PIK3CA, Mitogen-activated protein kinase 1 (MAPK1), AKT1, Phosphatidylinositol 4,5-bisphosphate 3-kinase catalytic subunit delta isoform (PIK3CD), Mitogen-activated protein kinase (MAPK3), GRB2, Epidermal growth factor receptor (EGFR), and SRC.

#### 3.3.5. Molecular Docking Results Analysis

**Figure 10:**
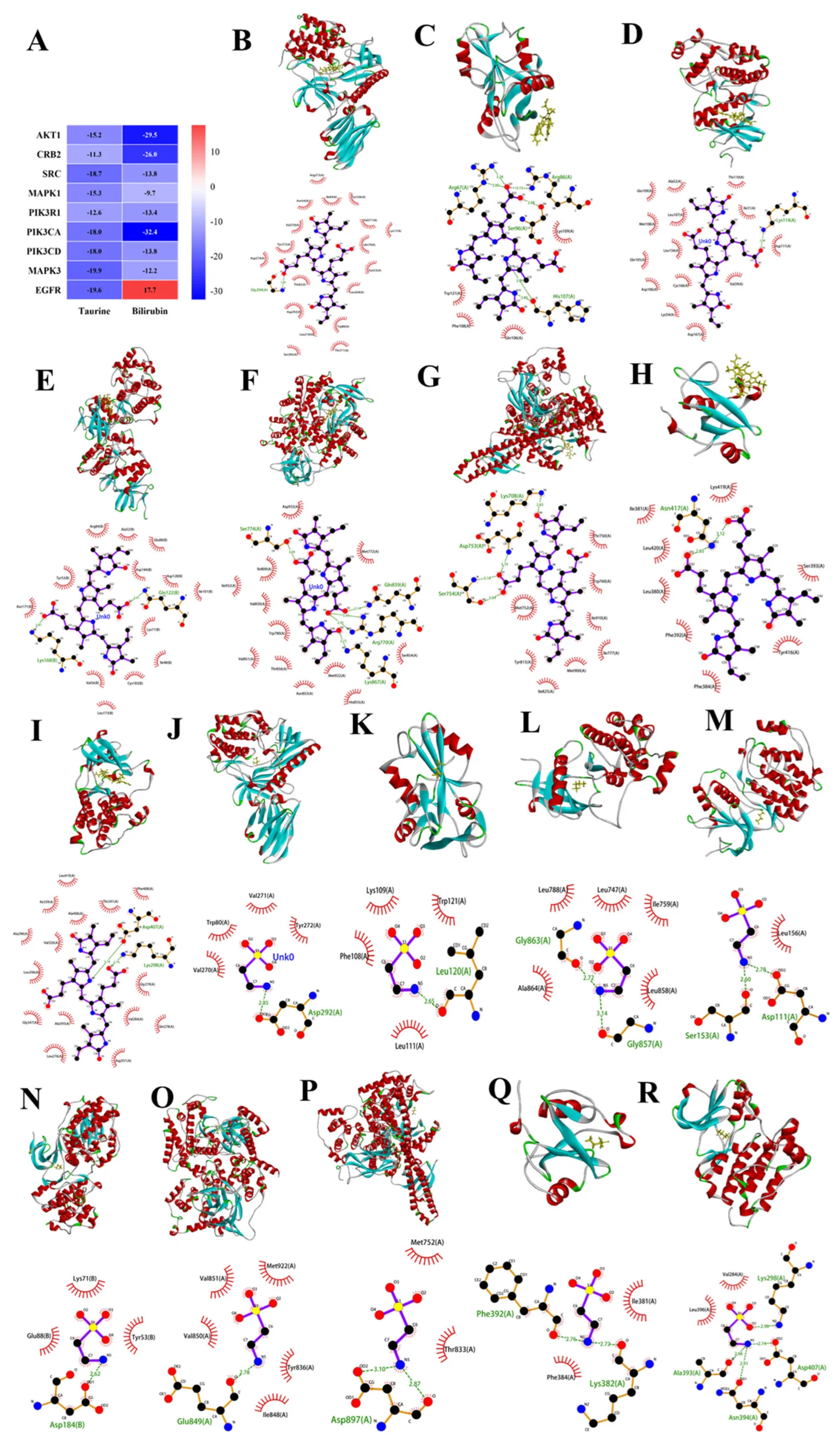
Molecular docking data and visual analytics. (**A**) Molecular docking data graph (kcal/mol). (**B**–**I**) Bilirubin with AKT1, GRB2, MAPK1, MAPK3, PIK3CA, PIK3CD, PIK3R1, and SRC, respectively. (**J**–**R**) Taurine with AKT1, EGFR, GRB2, MAPK1, MAPK3, PIK3CA, PIK3CD, PIK3R1, and SRC, respectively.

According to the key targets identified through topological analysis of the drug–component–target–disease network, PIK3R1, PIK3CA, MAPK1, AKT1, PIK3CD, MAPK3, GRB2, EGFR, and SRC were employed as receptors in molecular docking studies. The molecular docking results are shown in Figure 10. Taurine and bilirubin exhibited strong binding affinities with PIK3R1, PIK3CA, MAPK1, AKT1, PIK3CD, MAPK3, GRB2, and SRC, with binding energies all lower than −9.7 kcal/mol, suggesting that NBC and its substitutes may exert anti-seizure effects through these targets. In addition, taurine showed a strong binding affinity with EGFR, with a binding energy of −19.6 kcal/mol, indicating that EGFR may also be an important target mediating the anti-seizure effects of NBC and its substitutes.

#### 3.3.6. Potential Mechanisms Underlying the Anti-Seizure Effects of NBC and Its Substitutes

**Figure 11:**
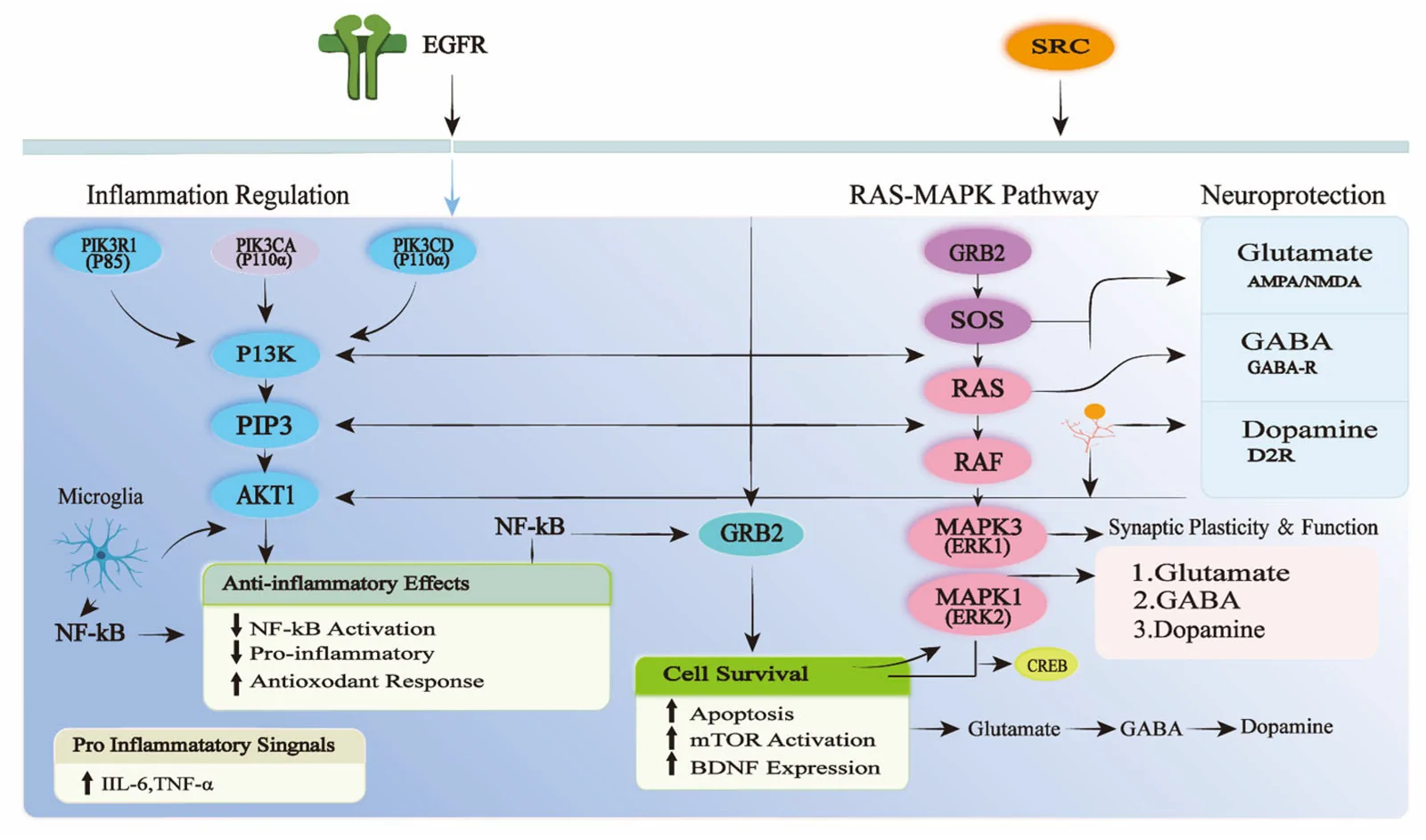
Potential mechanisms underlying the anti-seizure effects of NBC and its substitutes.

Additionally, signaling pathways associated with nine key targets were constructed and analyzed (Figure 11). The results show that PIK3R1, PIK3CA, PIK3CD, and AKT1 constitute the core of the PI3K–AKT signaling axis, whereas MAPK1 and MAPK3 are the key effector molecules of the MAPK/ERK pathway. EGFR, SRC, and GRB2 act as upstream signaling hubs that cooperatively activate and regulate both pathways, thereby achieving a dynamic balance of cell survival, inflammatory regulation, and proliferative repair through multi-level crosstalk and feedback mechanisms. In this process, inflammation regulation and neuroprotection are involved, which is consistent with animal studies demonstrating that NBC and its substitutes exert anti-seizure effects against PTZ-induced seizures through neuroprotection and modulation of neuroinflammation and neurotransmitter systems. These findings further support the notion that NBC and its substitutes exert anti-seizure effects through a synergistic multicomponent–multitarget-multipathway mode of action.

## 4. Discussion

Traditional Chinese medicine (TCM), which contains a variety of bioactive components, has a long history of treating epilepsy in diverse ways [11]. BCA is one of the major substitutes for NBC in clinical treatment, used for treating epilepsy and high fever [17,36]. The chemical library of BCA comprises bile acids, bilirubin, taurine, and other components [19,37]. Among these, bile acids, bilirubin, and taurine are proposed to be the primary bioactive compounds [16,17]. Since the first report on synthetic BCA in 1957, its anti-seizure effect has been demonstrated in studies that measured the incidence and recovery rate of seizures induced by cocaine and caffeine in mice [20]. Subsequently, Yu et al. reported that a new imitate Calculus Bovis exerted anti-convulsion effects against PTZ-induced seizure in mice [38]. Another study demonstrated that BCA prolonged seizure latency, reduced seizure frequency, and attenuated neuronal damage in rats with PTZ-induced seizures [39].

In this study, we systematically investigated the effects of different doses of BCA on PTZ-induced acute seizures in mice and evaluated the protective effects of NBC and its substitutes against PTZ-induced acute seizures, focusing on behavioral seizures, neuronal damage, and neuroinflammation. The results were consistent with previous reports and provided further evidence that NBC and its substitutes exert an anti-seizure effect.

More importantly, this study demonstrated that the protective effects of NBC and its substitutes against PTZ-induced acute seizures differ in both their molecular targets and their efficacies, likely due to differences in their chemical composition. Taurine, adenosine, bilirubin, bile acids, and some other compounds can be used as markers to discriminate NBC and its substitutes [19]. Taurine has been shown to ameliorate various neurological disorders, including epilepsy, and to protect the nervous system from injury and toxicity [40]. Adenosine is a potent endogenous anti-epileptic substance that can abolish seizure activity via the adenosine A_1_ G protein-coupled receptor [41]. Additionally, bilirubin exerts anti-seizure effects based on anti-inflammatory, anti-oxidant, modulating neurotransmitter systems, and so on [42]. Bile acids are potent modulators of the systemic pro-inflammatory/anti-inflammatory balance [43]. Furthermore, cultured bear bile powder has demonstrated anti-convulsant effects against febrile seizures via the regulation of neurotransmission and inhibition of neuroinflammation [44]. In summary, the protective effects of NBC and its substitutes against PTZ-induced acute seizures result from the combined action of multiple ingredients.

Chinese medicine acts against epilepsy through various mechanisms, such as anti-inflammation and neuroprotection, as well as modulating neurotransmitters [10]. Emerging evidence supports a direct relationship between neuroinflammation and seizures, suggesting that brain inflammation might play a key role in repeated seizures and behavioral comorbidities [1,8]. The chemical components in NBC and its substitutes have been reported to possess anti-inflammatory properties. For instance, taurocholic acid and glycocholic acid in Calculus bovis significantly inhibited the proinflammatory cytokines/chemokines in both in vivo and in vitro models [45]. Additionally, oral administration of taurine was shown to effectively alleviate heat stress-induced hippocampal inflammation [46]. In our study, treatment with NBC and its substitutes prolonged seizure latency and reduced the frequency, though their effects are not uniform. Simultaneously, the levels of IL-6, IL-1β and TNF-α in the hippocampus were reduced. Collectively, these findings support that seizures induce neuroinflammation, and NBC and its substitutes counteract protective effects against PTZ-induced acute seizures though its anti-inflammatory properties.

Seizures may develop due to a relative imbalance between excitatory and inhibitory neurotransmitters, which is one of the key mechanisms in seizure pathology [47]. It has been demonstrated that a series of neurotransmitter changes are linked to behavioral changes during PTZ-induced seizures [30]. Among these changes, GABA is a key driver in epilepsy and other neurological diseases due to its role as the central inhibitory neurotransmitter in the brain [48]. Taurine acts as a positive modulator on GABA_A_ receptors, thereby enhancing the anticonvulsive effects of muscimol and pentobarbital [49]. Additionally, taurine can exert anticonvulsant effects by modulating 5-HT and DA levels [50]. Our study results showed that NBC and its substitutes treatment could reverse the imbalance in 5-HT, GABA and DA levels caused by PTZ, thereby exerting an anti-seizure effect. Additionally, NBC and its substitutes treatment alleviated neuronal damage by reducing pathological changes and promoting neurogenesis in hippocampal. It has been reported that anti-seizure drugs are often accompanied by neuroprotective effects, anti-neuroinflammatory activity and modulation of neurotransmitters [51,52], which is consistent with our findings.

The role of PI3K–AKT signaling has been widely implicated in the pathogenesis of seizures [53,54]. In addition, it has been reported that the RAS/MAPK/ERK and P13K/AKT/mTOR pathways represent two major molecular alterations associated with seizures [55]. In this study, network pharmacology and molecular docking analyses were performed to explore the potential mechanism underlying the protective effects of NBC and its substitutes against PTZ-induced acute seizures. The predictions suggested that nine identified key targets were mainly concentrated in the PI3K–AKT signaling axis and the MAPK/ERK pathway. Through multi-level crosstalk and feedback mechanisms, these pathways collectively achieve a dynamic balance of cell survival, inflammatory regulation, and proliferative repair. Accordingly, it is hypothesized that NBC and its substitutes exert protective effects against PTZ-induced acute seizures through a comprehensive multicomponent, multitarget, and multipathway mode of action. This could potentially explain their universal protective effects against PTZ-induced acute seizures, despite their inconsistent regulation of neurotransmitters, inflammatory cytokines, and other related indicators.

In addition, this study has some limitations. First, we only employed the PTZ-induced acute model to compare the protective effects of NBC and its substitutes. This model is not considered a representation of drug-resistant seizures [56], and therefore, additional seizure models (e.g., MES, kindling, or chronic epilepsy models) are needed to further evaluate the protective effects of NBC and its substitutes. Second, we have not yet conducted systematic in vitro experiments prior to this in vivo study. In addition, the protective effects were evaluated using crude drugs in vivo, which lack chemical purity standards, and the specific compounds responsible for the observed activity were not identified. Third, taurine and bilirubin were selected for molecular docking because they are recognized as marker constituents of Bovis Calculus. However, other bioactive components, especially bile acids, may also contribute to the observed effects, possibly through synergistic interactions. These possibilities were not explored in the present study. Furthermore, the mechanistic insights regarding the PI3K–AKT and MAPK signaling pathways are primarily derived from network pharmacology and molecular docking predictions, which require further experimental validation in future studies.

## 5. Conclusions

This study provides a systematic comparative evaluation of the protective effects of NBC, BCS and BCA against PTZ-induced acute seizures using an integrated pharmacological assessment strategy. Although all three drugs exhibited protective effects of NBC, BCS and BCA against PTZ-induced acute seizures, their distinct neurochemical, inflammatory, and histopathological responses indicate that pharmacological similarity cannot be inferred solely from shared major constituents. These findings underscore the importance of multidimensional evaluation for distinguishing the therapeutic characteristics of closely related medicinal materials. More importantly, by establishing comparative evidence across behavioral, pathological, and biochemical dimensions, this work contributes to a more comprehensive understanding of the similarities and differences among Bovis Calculus sources. The results provide experimental support for the rational assessment of substitution potential and may facilitate the evidence-based utilization of alternative Bovis Calculus resources in future research and clinical practice. The present findings emphasize that the substitution of traditional animal-derived medicines should be supported not only by compositional similarity but also by comprehensive pharmacological evidence.

## Figures and Tables

**Table 1:** Major chemical constituents identified in BCA.

No.	Name	Molecular Formula
1	Bilirubin	C_33_H_36_N_4_O_6_
2	Biliverdin	C_33_H_34_N_4_O_6_
3	Chenodeoxycholic acid	C_24_H_40_O_4_
4	Cholesterol	C_27_H_46_O
5	Cholic acid	C_24_H_40_O_5_
6	Choline	C_5_H_14_NO^+^
7	Deoxycholic acid	C_24_H_40_O_4_
8	Glycochenodeoxycholic acid	C_26_H_43_NO_5_
9	Glycocholic acid	C_26_H_43_NO_6_
10	Glycodeoxycholic acid	C_26_H_43_NO_5_
11	Glycohyocholic acid	C_26_H_43_NO_6_
12	Glycohyodeoxycholic acid	C_26_H_43_NO_5_
13	Hyocholic acid	C_24_H_40_O_5_
14	Taurine	C_2_H_7_NO_3_S
15	Taurochenodeoxycholic acid	C_26_H_45_NO_6_S
16	Taurocholate	C_26_H_44_NO_7_S^−^
17	Taurocholic acid	C_26_H_45_NO_7_S
18	Taurohyodeoxycholic acid	C_26_H_45_NO_6_S
19	Taurolithocholic acid	C_26_H_45_NO_5_S

Note: The compounds listed are qualitatively identified.

## Data Availability

The original contributions presented in this study are included in the article. Further inquiries can be directed to the corresponding authors.
